# Interactome and reciprocal activation of pathways in topical mesenchymal stem cells and the recipient cerebral cortex following traumatic brain injury

**DOI:** 10.1038/s41598-017-01772-7

**Published:** 2017-07-10

**Authors:** Ping K. Lam, Kevin K. W. Wang, Anthony W. I. Lo, Cindy S. W. Tong, Don W. C. Ching, Kenneth Wong, Zhihui Yang, Themis Kong, Kin K. Y. Lo, Richard K. W. Choy, Paul B. S. Lai, George K. C. Wong, Wai S. Poon

**Affiliations:** 1 Department of Surgery, The Chinese University of Hong Kong, Hong Kong, SAR China; 2 Chow Tai Fook-Cheng Yu Tung Surgical Stem Cell Research Center, The Chinese University of Hong Kong, Hong Kong, SAR China; 3 Department of Anatomical & Cellular Pathology, The Chinese University of Hong Kong, Hong Kong, SAR China; 4 Department of Obstetrics and Gynecology, The Chinese University of Hong Kong, Hong Kong, SAR China; 5 Program for Neurotrauma, Neuroproteomics & Biomarkers Research, Departments of Psychiatry, McKnight Brain Institute, University of Florida, Gainesville, FL USA

## Abstract

In this study, GFP-MSCs were topically applied to the surface of cerebral cortex within 1 hour of experimental TBI. No treatment was given to the control group. Three days after topical application, the MSCs homed to the injured parenchyma and improved the neurological function. Topical MSCs triggered earlier astrocytosis and reactive microglia. TBI penumbra and hippocampus had higher cellular proliferation. Apoptosis was suppressed at hippocampus at 1 week and reduced neuronal damaged was found in the penumbral at day 14 apoptosis. Proteolytic neuronal injury biomarkers (alphaII-spectrin breakdown products, SBDPs) and glial cell injury biomarker, glial fibrillary acidic protein (GFAP)-breakdown product (GBDPs) in injured cortex were also attenuated by MSCs. In the penumbra, six genes related to axongenesis (Erbb2); growth factors (Artn, Ptn); cytokine (IL3); cell cycle (Hdac4); and notch signaling (Hes1) were up-regulated three days after MSC transplant. Transcriptome analysis demonstrated that 7,943 genes were differentially expressed and 94 signaling pathways were activated in the topical MSCs transplanted onto the cortex of brain injured rats with TBI. In conclusion, topical application offers a direct and efficient delivery of MSCs to the brain.

## Introduction

Traumatic Brain injury (TBI) is a leading cause of death and disability^1^. The outcomes depend on the extent of the primary injury and the sequel of the secondary injury which involve cerebral edema, hematomas, hydrocephalus, impaired systemic and cellular metabolism, excitotoxicity and intracranial hypertension^2, 3^.

Pharmacological agents target a single pathophysiological mechanism but TBI is a highly complex pathological disorder. To date, there are no effective neuroprotective pharmacological agents to reverse the sequels of TBI on either cellular or sub-cellular level. Mesenchymal stem cells (MSCs) are typically able to self-renew and differentiate into diverse somatic lineages to repopulate the damaged tissue. The ease of isolation from various tissues and rapid *ex vivo* expansion make MSCs prime candidate for tissue engineering and therapeutic applications. Although there are concerns that MSCs may undergo spontaneous formation after long-term (four to five months) culture^4^, infusion of expanded MSCs with less than six to eight weeks in culture 10 is considered safe^5^. No toxicity related to MSC transplant has been reported. Many preclinical studies have shown that differentiation of MSCs into neuronal cells is not considered as the major recovery mechanism in the context brain injury because of low engraftment of MSCs into brain parenchyma^6^. Neurological benefits of MSCs may be attributed by paracrine and cytokine actions. MSCs secrete neurotrophic factors that enhance angiogenesis, proliferation of endogenous neural stem cells and neuroprotective factors that suppress neuro- inflammation and apoptosis^7, 8^.

Therapeutic potentials of MSCs are commonly investigated by administrating MSCs through systemic infusion or direct injection into the brain in animal experiment or human studies. By borrowing concept from our tissue engineering technology previously developed for transplantation of cultured epidermal skin graft to burn wound/chronic wounds^9^, we have developed a novel technique to deliver a large amount of MSCs directly to the target organs^10, 11^. It is our hypothesis that when MSCs are topically applied to the surface of recipient organs, the MSCs can home to the injured parenchyma. Our previous experiment showed that topically applied MSCs could migrate from the surface of cerebral cortex in the contralateral side to the penumbra of TBI in the ipsilateral cerebral hemisphere, apparently following pre-existing axons along the corpus callosum^11^.

To maximize the therapeutic potential of MSCs in this study, we topically applied MSCs to cerebral cortex of the TBI site. The interactome and reciprocal activation of pathways in the topical MSCs and recipient cortex were also studied.

## Results

### MSC characterization

**Figure 1:**
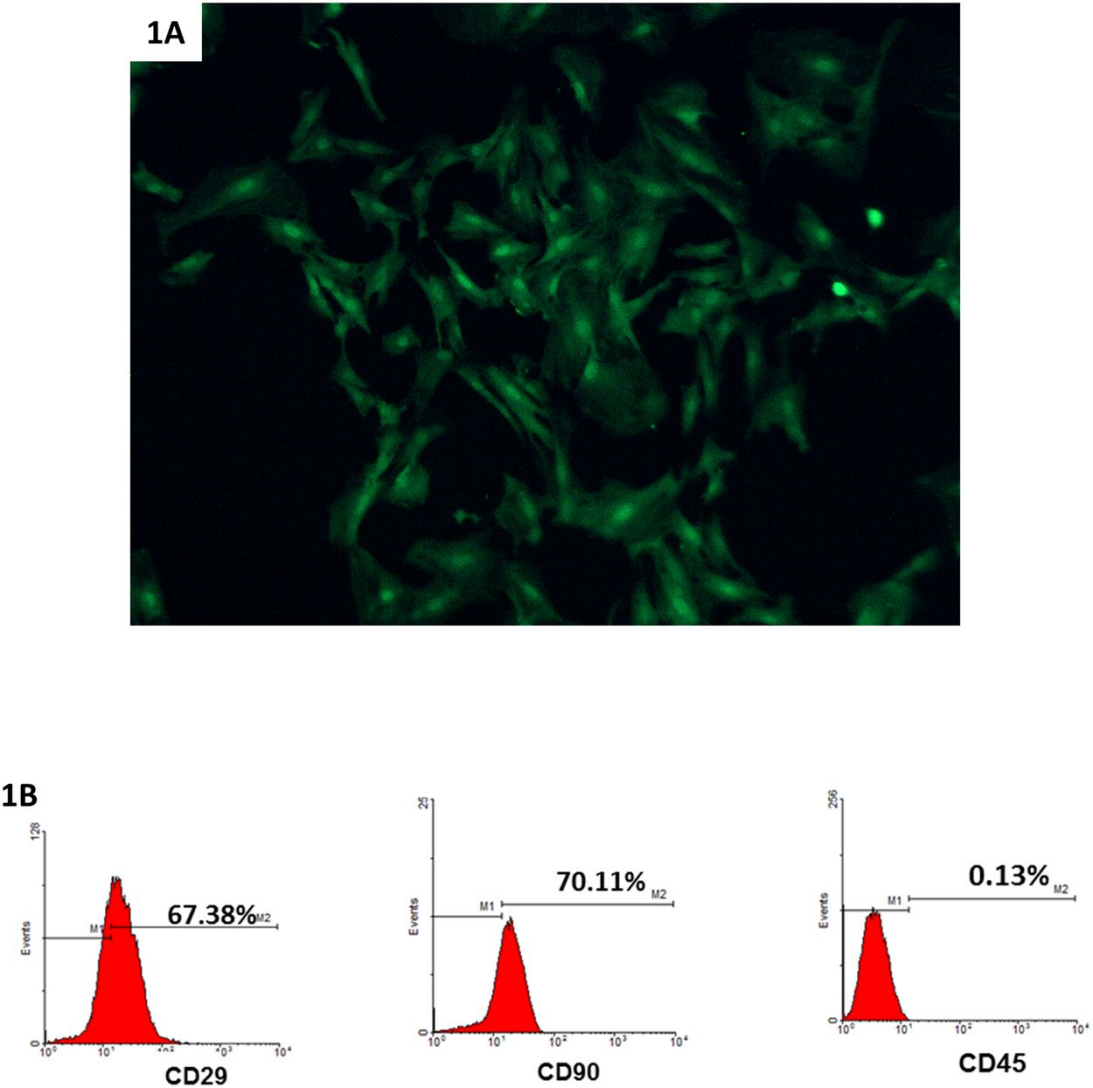
MSC phenotype and characterization in culture. An image of GFP-MSCs in culture showed a spindle-shaped morphology (**1A**), phase-contrast microscopy, x100). Flow cytometric analysis of MSCs using phycoerythrin-conjugated anti-CD29, anti-CD90 and anti-CD45 (**1B**).

TheMSCs isolated from adipose tissue of transgenic GFP-SD rats were adherent to the plastic culture flasks and exhibited spindle-shape morphology (Fig. 1A). They demonstrated *in vitro* differentiation potential into adipocytes, chrondroblasts and osteoblasts under specific differentiating environments (data not shown). Flow cytometric analysis demonstrated that the MSCs expressed CD29 and CD90, and were negative for CD45 (Fig. 1B).

#### Histology and Immunohistochemistry of rat cortex following TBI with and without MSC transplantation

**Figure 2:**
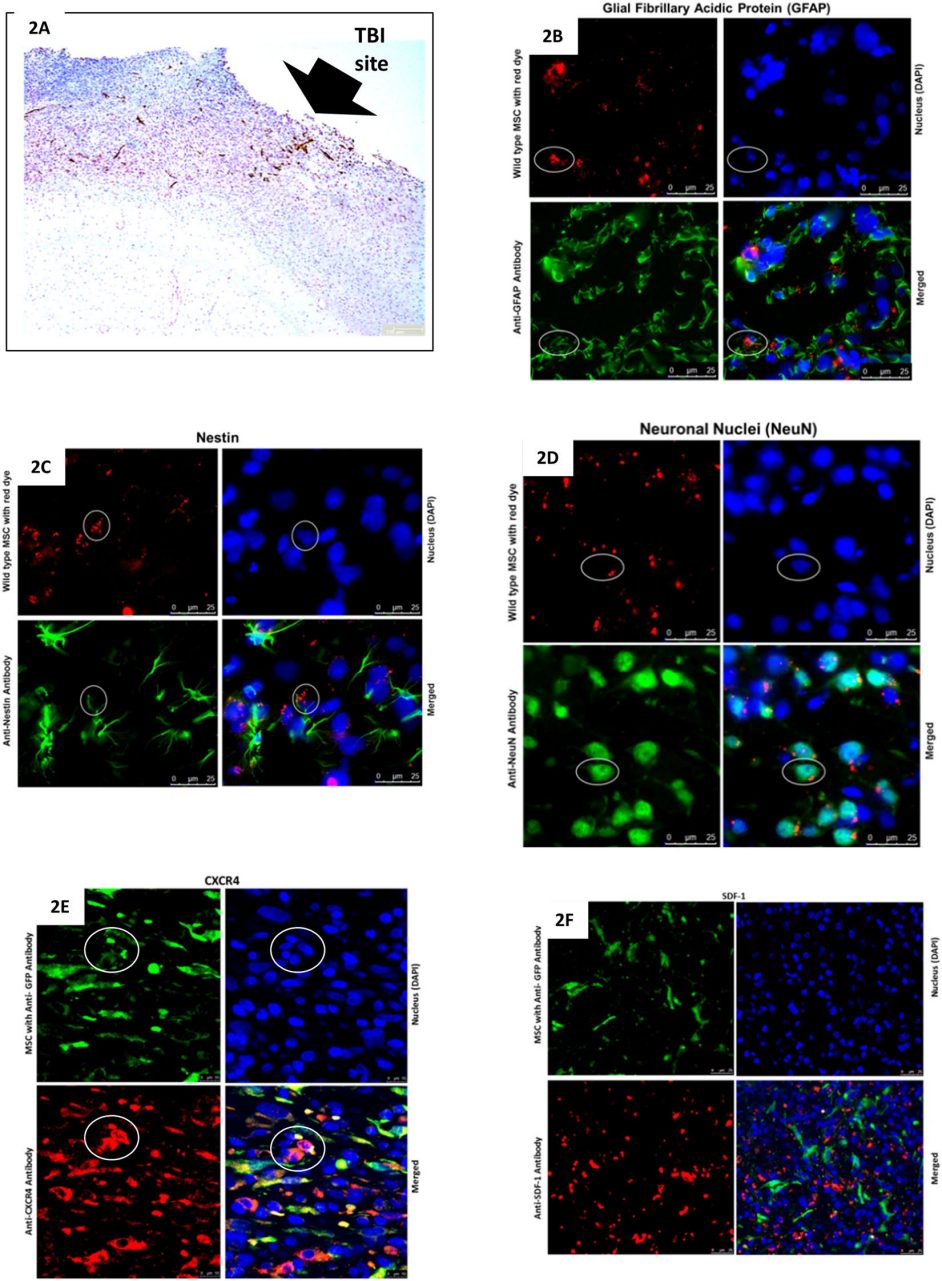
Fate of GFP+ve MSCs transplanted onto penumbra cortex following TBI. Few GFP+ve cells (arrows) were found in the penumbral region of TBI 3 days after topical application. (**2A** Immunohistochemistry staining IHC x200; **2B** H&E x200). A TBI lesion without treatment (**2C** H&E x200). The homed MSCs which were pre-labeled with CM-DIL red fluorescence dye expressed markers of GFAP (Green) (**2D** Immunofluorescent staining IF x200); Nestin (Green) (2E IF x200) and NeuN (Green) (**2F** IF x200). CXCR4 (Red) was expressed by the homed MSCs (Green) (2 G IF x200). In the vicinity of homed MSCs (Green), the TBI penumbra expressed SDF-1 (Red) (2 H IF x200). DAPI (Blue) was used to stain the nucleus.

Three days after topical application, most of the MSCs proliferated at the site of application. Few MSCs (less than 0.1%) migrated from surface of cerebral cortex to the penumbra of brain injury (Fig. 2A–C). These cells co-expressed GFAP (a marker of astrocyte) (Fig. 2D) and neuronal markers (Nestin, NeuN) (Fig. 2E,F). Compared with contralateral hemisphere, ipsilateral cerebral cortex expressed stromal cell-derived factor-1(SDF-1) where the MSCs homed (Fig. 2G). The chemokine receptor, CXCR4 was expressed by MSCs homed in the penumbra (Fig. 2H).

**Figure 3:**
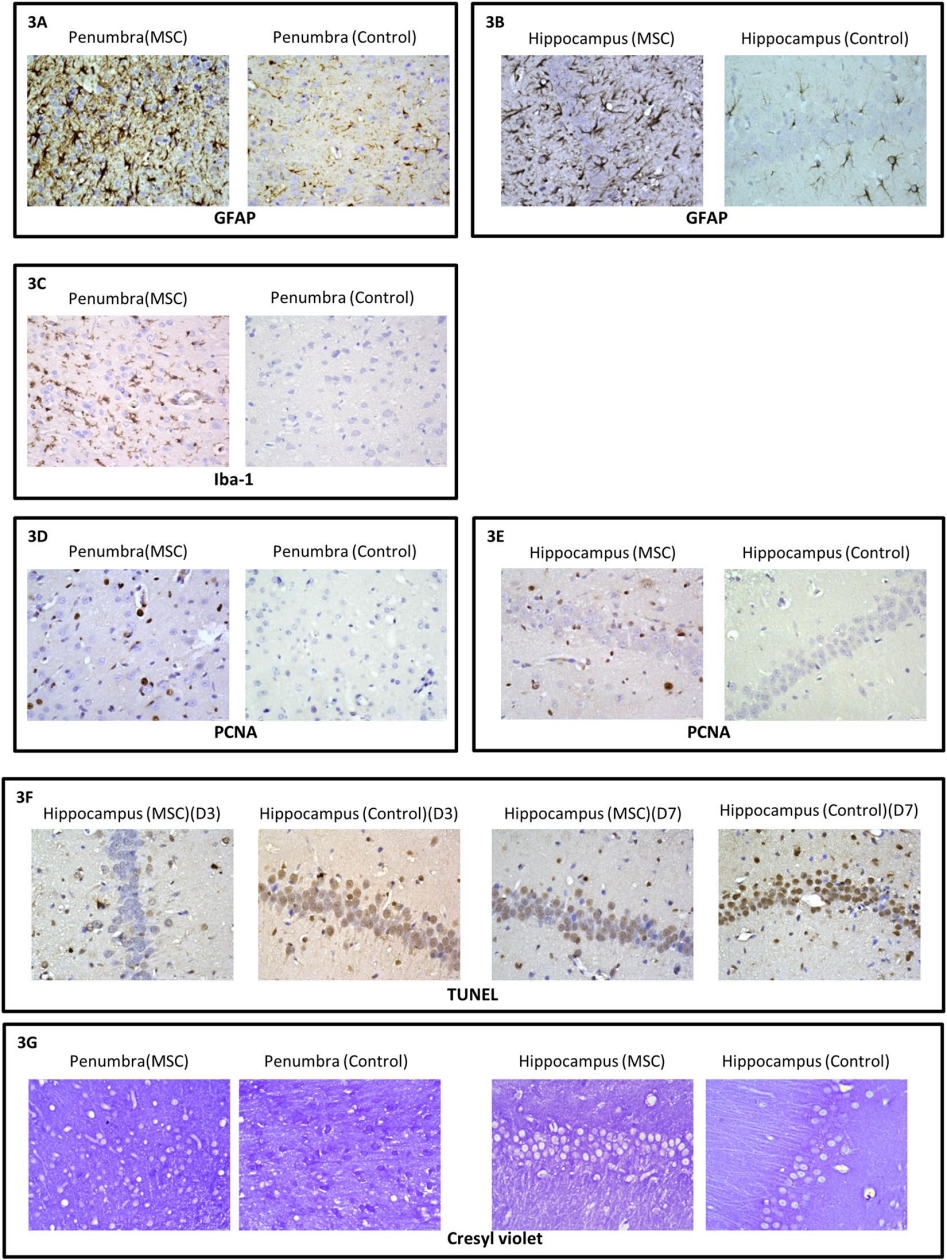
Representative photos of histochemical staining images at different time points post-MSC treatment. At day 3, MSC-treatment group had more GFAP+ve cells in the TBI penumbra (**3A**, IHC x400) and hippocampus (**3B** IHC x400). More Iba-1+ve cells (**3C** HIC x400) were observed in the penumbra of MSC-treatment group. Both the penumbra (**3D** IHC x400) and hippocampus (**3D** IHC x400) also had higher PCNA expression. MSCs attenuated apoptosis in the hippocampus (**3F** Tunel, x400). At day 14, more neurons were found in both penumbra and hippocampus of MSC-treatment group (**3G** Cresyl Violet x400).

The MSCs triggered earlier astrocytosis and reactive microglia at day 3 with more GFAP-positive cells in both penumbral cortex (Fig. 3A) and hippocampus (CA1/CA3) (Fig. 3B), and more Iba1-positive cells (a marker of microglia) in penumbra (Fig. 3C) (p < 0.05). Higher cellular proliferation characterized by PCNA expression was found in penumbra (Fig. 3D) and hippocampus (Fig. 3E). Moreover, topical MSCs attenuated apoptosis in the hippocampus at days 3 and 7 (Fig. 3F). At day 14, Cresyl violet staining showed that there was a lower degree of neuronal death in penumbra and hippocampus (Fig. 3G; Table 1). No GFP+ve cells were found in the brain parenchyma from day 7 after topical application (Data not shown).

### Behavioral tests

**Figure 4:**
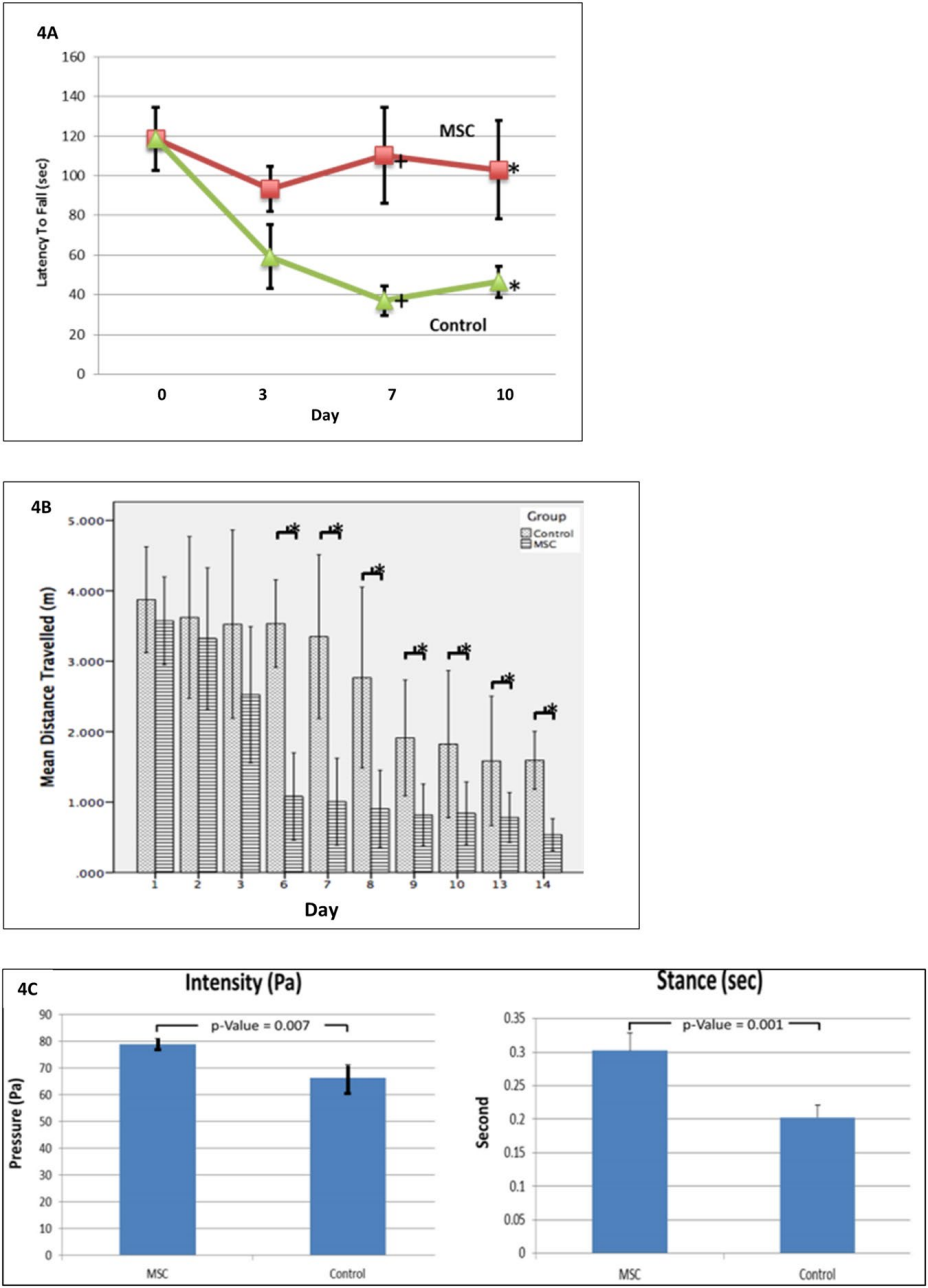
Behavioral assessment of rats with and without MSC treatment following TBI. Rats treated with topical MSCs showed a significant improvement in behavioral assessment. Parameters were expressed in mean + standard deviation. Topical MSCs improved the coordination and integration of movement motor function in the Motor Rod Test (**4A**). The animals travelled a shorter distance to reach the platform in the Water Maze Test (**4B**). Automated gait analysis illustrated the greater intensity (mean pressure exerted on the floor by one individual paw) and longer stance (duration during which the paw is in contact with the glass plate) by the MSC-treatment group (**4C**).

The balance and motor function at days 3, 7, and 10 were improved in the MSC-treated rats. The latency to fall from the accelerating motor rod was significantly longer (p < 0.05) (Fig. 4A). The spatial learning and memory were also rescued. The rats travelled a shorter distance to reach the hidden platform in Morris Water Maze assessment at days 6 to 14 (P < 0.05) (Fig. 4B) and exerted greater pressure on the glass plate with longer contact duration when crossing the walkway at day 3 in the gait analysis study (Fig. 4C) when compared to the CCI counterpart (p < 0.05).

### Brain injury biomarker analysis of the injured rat cortex

**Figure 5:**
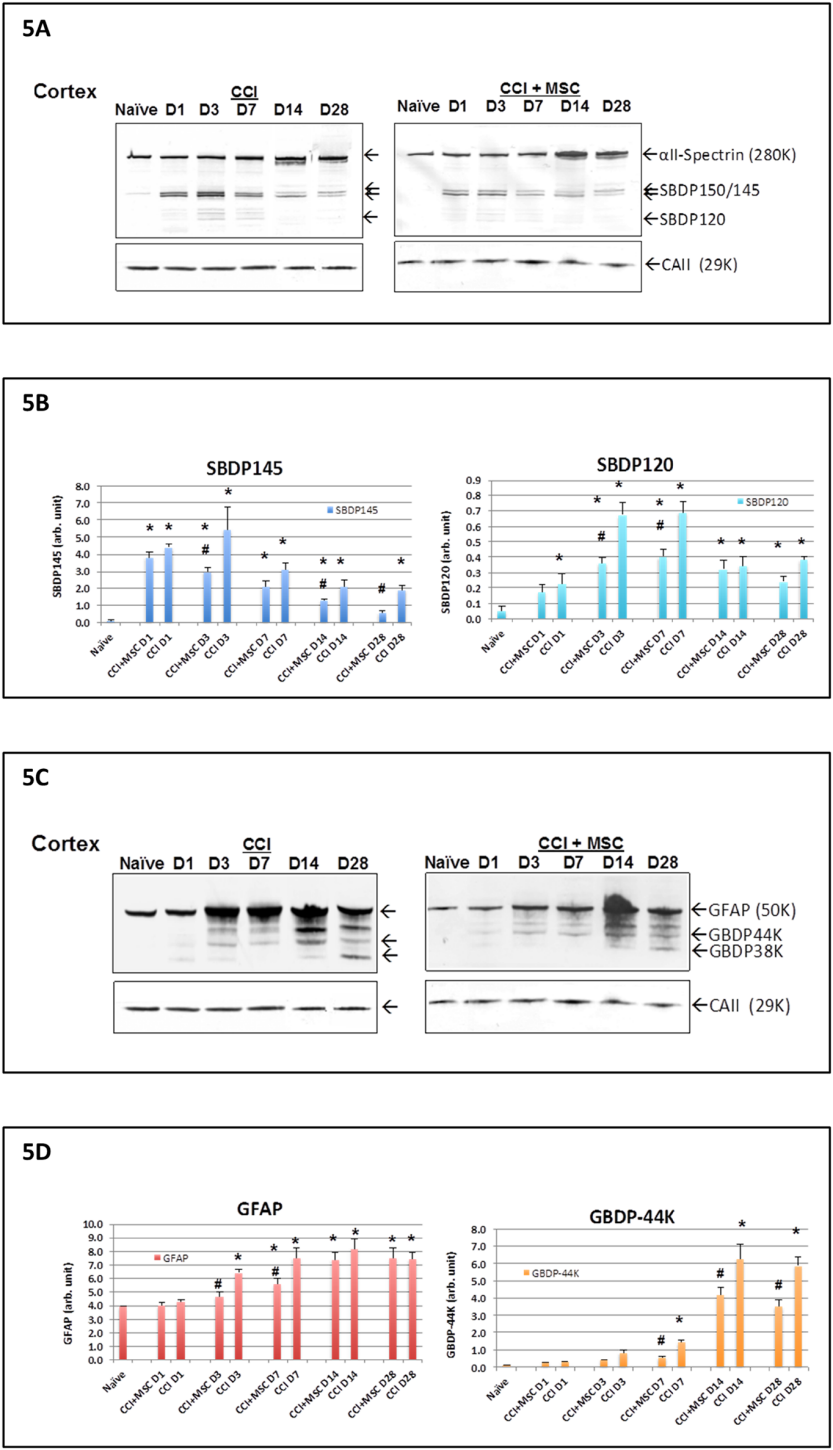
Expression of biomarkers in penumbra cortex in rats with and without MSC treatment after TBI. Effects of MSC topical transplantation on proteolytic neuronal and glial injury biomarker levels in the ipsilateral cortex axonal alphaII-spectrin breakdown product SBDP biomarkers levels at different time points after controlled cortical impact (CCI) injury with or without MSC topical transplant are shown. (**A**) showed representative blots of spectrin and house-keeping gene loading control (Carbonic anhydrase II, 29 kDa). (**B**) showed quantification of biomarkers (equalized by CA-II band intensity). GFAP and GFAP-breakdown product GBDP biomarkers levels at different time points after controlled cortical impact (CCI) injury with or without MSC topical transplant were shown (**C**,**D**) shows representative blots of GFAP and house-keeping gene (Carbonic anhydrase II, 29 kDa). Quantification of comparison: *higher than naïve group; ^#^MSC + CCI treatment group was different from CCI alone group at same time point (p < 0.05). MSC + CCI vs. CCI group-based ANOVA results were stated in the text.

Neuronal and axonal injury biomarker alphaII-spectrin breakdown products- SBDP150 and SBDP145 (generated by pro-necrosis calpain) levels were elevated early (day 1–14), while SBDP120 (generated by post-apoptosis) caspase-3 levels were elevated in a delayed fashion (day 3–28) in the ipsilateral cortex after controlled cortical impact injury. Importantly, both SBDP145 and SBDP120 levels were attenuated in the CCI+MSC group as compared to the CCI group by ANOVA test with all post-injury time points (day 1, 3, 7, 14 and 28) taken into account (Fig. 5A,B). In parallel, glial injury biomarker GFAP- breakdown products (e.g. GBDP-44K) also appeared after CCI in a further delayed fashion (day 7–28). Again, the CCI+MSC group has significantly attenuated GBDP levels with all-time points included for ANOVA analysis (Fig. 5C,D). Further pair-wise Tukey tests also found that SBDP145, SBDP120 and GBDP-44K were significantly lower at several points after injury, respectively (Fig. 5B,D). Lastly, intact GFAP, which is a marker of reactive gliosis, was induced at day 3 through 28 after CCI, with GFAP suppression observed only at day 3–7 by MSC treatment.

### Micro-array analysis of MSC layer following transplantation to injured rat cortex

**Figure 6:**
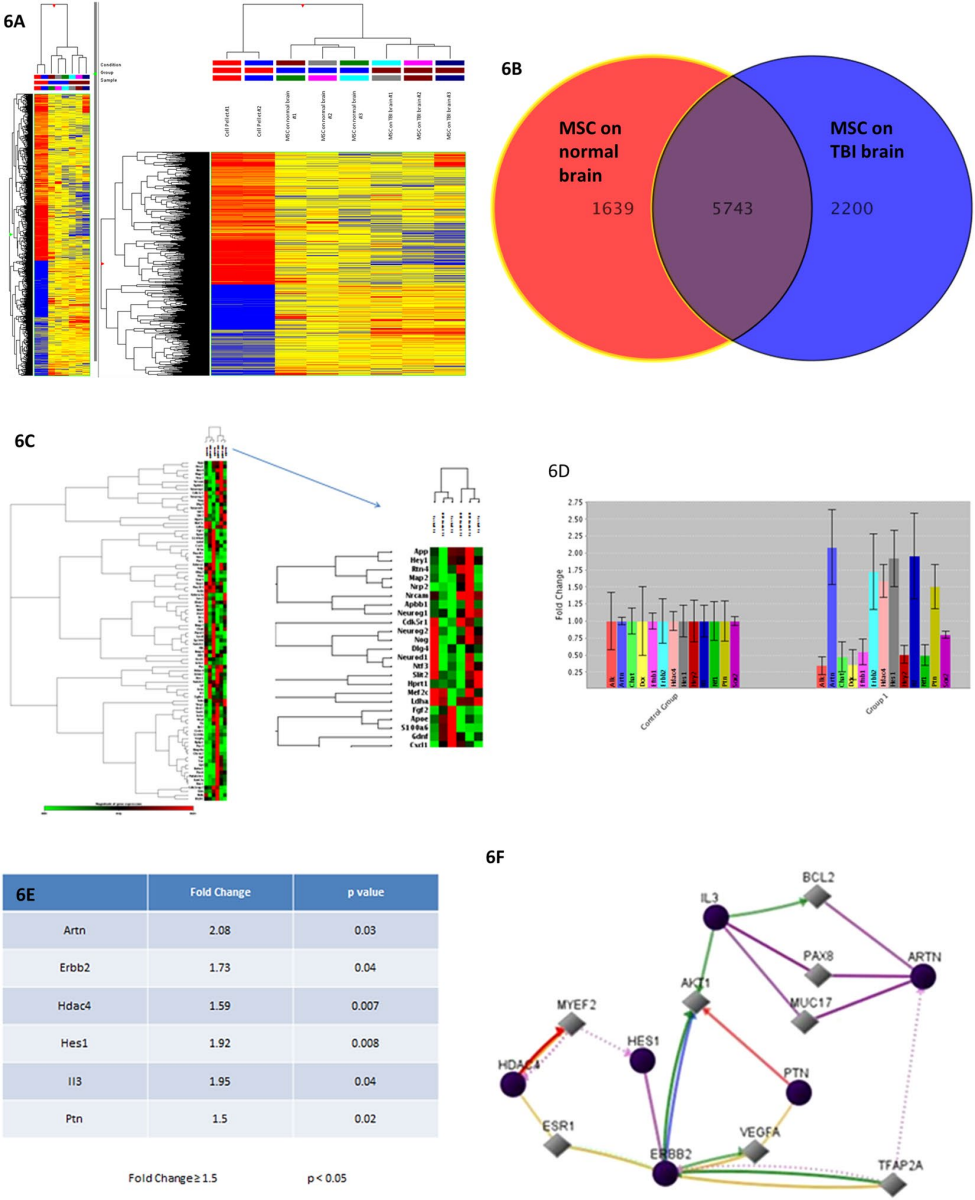
Analysis of gene of MSC and penumbra cortex at day 3 following TBI. Hierarchical clustering of gene expression of MSC pellets before and after topical application on TBI brain and normal brain. Gene lists were generated by comparing MSC pellet versus topical MSCs on TBI brain and topical MSCs on normal brain using moderate T-test and 2-fold filtering in GeneSpring. Down-regulated genes were shown in blue and up-regulated genes were shown in red (**6A**). Venn diagram illustrated the number of genes which were up- or down-regulated of topical MSCs on TBI brain and normal brain using moderate T-test and 2-fold filtering in GeneSpring. Overlapping area indicated genes from MSCs pellets were coherently altered in both conditions (**6B**). Cluster analysis of PCR-array on 84 genes associated with neurogenesis in penumbral cortex treated topical MSCs (**6C**). Compared with control, 13 genes were significantly up-regulated (p < 0.05) (**6D**). 6 out of 13 genes were up-regulated with equal or more than 1.5 fold (**6E**). Diagram of the six-gene network which was proposed to get involved in the neurogenesis mediated by the topical MSCs. Grey color genes participated in the network but they were not detected by the PCR-array (**6F**).

Three days after topical application, the fibrin layer containing the MSC population was peeled off from the brain cortical surface and the transcriptome of MSC was studied using micro-array with probes for more than 35,000 genes. We compared the whole genome expression profile of MSCs staying on the cortical surface of TBI brain with the prolife of MSC population on normal uninjured brain, while using “control MSCX pellet” as reference. Genes were considered significant differentially regulated when the change >2-fold up or down regulated and p < 0.05 in MSC population on TBI brain when compared to MSC population on normal brain. Hierarchical cluster analysis demonstrated 7,943 genes significant differentially expressed. (Fig. 6A) These differentially expressed (DE) genes were annotated in Gene Ontology (GO) using GeneSpring 12.6. They covered a wide spectrum of genes related to organic substance metabolism (22.1%); protein metabolism (14.3%); gene expression (13.9%), response to stress (11.6%), and cellular macromolecule biosynthesis (10.6%), etc. Subsequent Venne Diagram analysis revealed 2,200 DE genes were unique in the MSC population growing on the surface of brain with TBI, whereas 5,743 DR genes were commonly found in both MSCs population growing on TBI brain and on normal brain. (Fig. 6B) The changes of expression of 16 DE genes known to affect acute inflammatory response, protein metabolic process were found consistent with RT-PCR validation. (Table 1) Pathways were analyzed by Genespring GX based on the database in Wikipathway and BioPAX. We then searched for genes which were overlapping within the test pathway with significant p-value. In this experiment, 94 pathways were significantly activated in the MSCs population on cerebral cortex with TBI (p < 0.005) (Table 2).

**Table 1:** Validation of micro array data with RT-PCR- *P < 0.05.

Ontology	Gene	Micro-array		RT-PCR	
		Fold Change	Regulation	Fold Change	Regulation
Protein-lipid Complex	Cd36	1134.63*	up	121.92*	up
Phospholipid efflux	Abcg1	112.78*	up	41.84*	up
Protein metabolic process	Cd74	11294.02*	up	1118.68*	up
	Aif1	7271.00*	up	1775.50	up
Extracellular region	Napsa	4238.69*	up	47.39*	up
Response to stress (includes acute inflammatory response, inflammatory response, defense response)	Cd74	11294.02*	up	1118.68*	up
	Aif1	7271.00*	up	1775.50	up
	S100a9	6325.14*	up	1.35	up
	Ctss	5313.30*	up	189.43*	up
	C1aq	2920.44*	up	2010.42*	up
	Itgb2	2851.24*	up	950.36	up
Ribonucleoprotein complex	Bco2	30.42*	up	6.02*	up
Enzyme regulator activity	Nos2	590.80*	up	3335.02*	up
	Ccl3	534.34*	up	161.36*	up
Lipid binding	Trem2	3158.37*	up	162.69*	up
Steroid metabolic process	Abcg1	112.78*	up	41.84*	up

**Table 2:** Most significantly activated twenty pathways in the MSC population topically applied on TBI brain, using MSC pellet as reference.

Pathway	p-value	Matched Entities	Pathway Entities of Experiment Type
Rn_T_Cell_Receptor_Signaling_Pathway_WP352_69416	7.27E-17	71	129
Rn_Type_II_interferon_signaling_(IFNG)_WP1289_69366	1.94E-11	26	34
Rn_Kit_Receptor_Signaling_Pathway_WP147_69456	3.47E-10	39	67
Rn_B_Cell_Receptor_Signaling_Pathway_WP285_67073	9.64E-10	68	155
Rn_Myometrial_Relaxation_and_Contraction_Pathways_WP140_71831	1.12E-08	67	155
Rn_IL-4_Signaling_Pathway_WP182_69413	4.05E-08	32	58
Rn_Calcium_Regulation_in_the_Cardiac_Cell_WP326_72154	6.45E-07	61	149
Rn_Insulin_Signaling_WP439_69418	1.11E-06	61	157
Rn_Toll-like_receptor_signaling_pathway_WP1309_72183	2.11E-06	40	91
Rn_IL-5_Signaling_Pathway_WP44_69358	2.48E-06	33	68
Rn_IL-3_Signaling_Pathway_WP319_69359	2.95E-06	43	101
Rn_Adipogenesis_WP155_69397	3.01E-06	52	130
Rn_Inflammatory_Response_Pathway_WP40_71795	7.81E-06	18	30
Rn_IL-6_Signaling_Pathway_WP135_69356	1.11E-05	41	97
Rn_NFE2L2_WP2376_71118	1.49E-05	59	161
Rn_GPCRs,_Other_WP409_71799	1.72E-05	32	75
Rn_Complement_and_Coagulation_Cascades_WP547_69384	2.15E-05	27	62
Rn_G_Protein_Signaling_Pathways_WP73_71295	4.13E-05	38	91
P2Y receptors	5.70E-05	8	11
Rn_Glutathione_metabolism_WP469_70700	5.70E-05	8	38

### PCR-array analysis of rat cortex following TBI with or without topical MSC treatment

The expression of 84 genes commonly associated with neurogenesis was studied using PCR array at day 3 (Fig. 6C,D). There were 6 DE genes (fold change >1.5, p < 0.05) (Fig. 6E) in the post-TBI penumbral cortex treated by topical MSCs, compared with the penumbral cortex without MSC treatment as counterpart. These genes were related to axonogenesis (Erbb2); growth factors (Artn, Ptn); cytokine (IL3); cell cycle (Hdac4); and notch signaling (Hes1). Using the Gene Network CentralTM provided by Qiagen, a molecular pathway network was predicted (Fig. 6F).

## Discussion

TBI is a complex neurological disease. The primary brain injury is the initial physical insult to the parenchyma that cannot be treated. The complexity of secondary brain injury involves brain edema, hydrocephalus, elevation of intracranial pressure, and hemorrhage into brain tissue, activation of resident glial cells, microglia and astrocytes, and infiltration of blood leukocytes, resulting in neuronal and glial death as well as traumatic axonal injury^12^. TBI in human causes neuronal death in the regions of cortex, hippocampus, cerebellum and thalamus. In rodent models of TBI, neuronal degeneration is evident in the cortex and hippocampus^13^. This current experiment demonstrated the homing potential of topically applied MSCs which improved the functional outcome following TBI. More than 7,900 genes of the topical MSC population were differentially expressed. Six genes related to axongenesis, growth factors, cytokine, cell cycle and notch signaling were significantly up-regulated in the penumbra cortex treated with topical MSCs.

Central nervous system has only minimal intrinsic regenerative capabilities to regenerate injured tissue in neurodegenerative and acute neurological disorders because the small number of residential stem cells in the brain does not significantly contribute to the complete functional recovery. There is no effective pharmacological treatment to confer neuroprotection by targeting the complex secondary injury pathophysiology. MSCs characterized by prolonged self-renewal and multi-lineage differentiation capabilities. In contrast to pharmacologic agents, MSCs are believed to function through multiple mechanisms to target the inflammatory and immunologic pathways. MSCs thus hold great promises for repair and regeneration of the injured brain tissue. Previous studies have shown that MSCs engrafted in animal models of brain injury and ameliorated neurological deficits^14–16^.

Delivery of MSCs to the brain is a prerequisite for the maximal execution of the neuro-protective effects. MSCs were either administrated by intravenous/intra-arterial (2 − 4 × 106) or intracerebral (1 − 6 × 106) injection in experimental models of TBI^17–20^. Systemic infusion of expanded MSCs to patients is considered safe. This route of administration is commonly adopted in most clinical trials as a large amount of MSCs can be transplanted by infusion. However, the homing of infused MSCs is hindered by the blood brain barrier (BBB) unless it is severely damaged following TBI to allow the passage of MSCs. The mean diameter of MSCs is smaller than that of pulmonary capillaries which trap the majorities of the infused MSCs^21^. This is one of the reasons for the inconsistent outcome in the clinical trial of MSCs^22^. Retention of large amount of exogenous MSCs in the lung may cause unfavorable complications. The threat of arterial embolism and occlusion also limit intra-arterial administration of MSCs. Intracerebral injection of MSCs into the brain is risky as evident from the unfortunate young boy who died after receiving a direct implantation of stem cells through Hamilton syringe^23^. Moreover, Cell suspension has to be gently triturated through small needles to keep MSCs dispersed and free of cell clumps. These repeated triturations through the syringe needles inevitably damage the cell membranes and adversely affect the MSCs viability and subsequent engraftment. Although ketamine has been found to be neuroprotective in models of cortical injury and ischemia, it should not affect the results of the behavior tests because same dosage of ketamine was administrated in both test and control groups.

Nowadays, tremendous research in the area of regenerative medicine is focused on the development of an effective transplantation technique for stem cells. One of the MSCs characteristics is their preferential homing to sites of tissue damage or inflammation. Chemokine receptors and their chemokine ligands are essential factors mediating the migration of leukocytes into sites of inflammation; it is possible that they are also involved in migration of other types of progenitor cells^24^. Compared with contralateral hemisphere, SDF-1 protein expression was up-regulated in the inflammatory ipsilateral side of TBI. In the present study, there were no MSCs in the contralateral hemisphere. CXCR4 is the cell surface receptor of SDF-1 and the expression of all chemokine receptors in general is very low^25^. The MSCs found in the penumbra expressed CXCR4. Thus, it is probable that SDF-1/CXCR4-axis was involved in the trafficking of topical MSCs to the penumbra.

Previous studies showed that exogenous MSCs modulated inflammatory response through down-regulation of pro-inflammatory cytokines and up-regulation of anti-inflammatory factors; and secreted various growth hormones such as VEGF and FGF to mediate angiogenesis and vascular stabilization in penumbra of TBI. In addition, neurogenesis in the SGV and SVZ was enhanced, glial scar formation was suppressed^26^. Topical transplantation of cells is conducted primarily in dermatology. To best of our knowledge, we were the first group to transplant MSCs to the brain by topical application. Our data could not be compared with the literatures directly. In this study, only few (less than 0.1%) topical MSCs homed to the cerebral cortex and expressed markers of astrocyte and neuron that was too few to account for the improvement of functional outcome after TBI by the mechanism of differentiation. The acute functional recovery as demonstrated in the motor rod and gait analysis precluded the neuro-repair through differentiation of MSCs because it was unlikely for MSCs to migrate from the surface of cerebral cortex into the injured site and undergo a complete differentiation to replace the impaired neurons within 72 hours. The engrafted MSCs had a short survival and no viable MSCs were detected in the brain parenchyma from day 7 after topical application. But the neuro-protective effects were sustained up to 14 days as demonstrated in motor rod test and gait analysis. Topical application of MSCs increased the PCNA expression as well as the number of astrocytes and microglia in acute/sub-acute phage of brain injury. These cells were not over-activated to cause astrogliosis as the number of these cells decreased by day 14. We interpreted this result as the MSC induced an overall brain cell proliferation as a result of a possible trophic response. But we saw no evidence of tumor formation in all the rats that received MSC treatment’s in our study. Taken together, it is believed that topical MSCs enhanced the neuro-repair not via differentiation but rather via complex paracrine actions. Although some groups suggested that some stem cells grafting could result in cerebral microbleeding, which might trigger inflammatory response and could augment the host reaction against the graft, this was not considered in this current study because only a few MSCs were found in the injured brain parenchyma and they had a short survival. Higher cellular proliferation characterized by PCNA expression was found in penumbra and hippocampus. We interpreted this result as the MSCs induced an overall brain cell proliferation. But we saw no evidence of tumor formation in all the rats that received MSC treatment’s in our study.

Most of the data on stem cell signaling pathway are based on *in vitro* studies because it is unlikely to harvest the transplanted stem cells from recipient organs for molecular analysis. Topical application offers an additional advantage that the MSCs could be harvested from the recipient surface of cerebral cortex after the exposure to the microenvironment of TBI. For the first time, the activated signaling pathways of topical MSCs on the brain surface were reported in experimental TBI. This information is useful for further investigation of gene therapy of stem cells. On the other hand, the beneficial roles of topical MSCs that do not home to the site of injury should not be overlooked. If these MSCs on the surface of brain released paracrine and/or endocrine factors, topical application could offer an additional therapeutic potential because these biofactors would be directly delivered to the brain or at least produced in sites close to the target organ. In fact, the signaling pathways of the topical MSCs on the brain surface were significantly activated in this study. There were six DE genes related to axonogenesis, growth factors, cytokines, cell cycle and notch signaling in the penumbra. 2,200 unique DE genes were detected in the topical MSCs population residing on the surface of the cerebral cortex. The underlying molecular interaction between this MSCs population and the underneath brain parenchyma remains to be determined.

### Limitation of the current study

In this study, we have showed that brain protein biomarker reduction is responsive to therapeutic effect of MSC intervention (e.g. Fig. 5; MSC vs. Non – MSC treatment). Similarly, we have shown that with MC topical treatment, a distinct set of genes with alerted expression levels can be identified in the recipient rat brain. Ideally, one should directly examine if there are correlations between the two. However, the tissue samples used for immunoblotting followed by SBDP/GBDP quantification (protein biomarker analysis) were from a set of rats that is distinct from the tissue set we used for gene profiling/signaling pathways identification (mRNA analysis). This is because the tissue preparations for protein/mRNA analysis are incompatible and the brain tissue amount in one animal is not sufficient for both types of analysis. Thus, we could not perform same-animal correlation between biomarker reduction and alerted gene expression or pathways.

Lastly, exploring the molecular biology evidence or basis for behavioral improvements (such as activity-induced genes) is potentially an important concept. However, due to multiple gene/signal pathways that are involved, it will require an expanded study design, such as the use of gene knockout or knockdown experiments. These studies are beyond the scope of our current study; nonetheless, they should be pursued as future area of research.

In conclusion, the simplicity of the topical application makes it a very attractive procedure in clinical setting to deliver a large amount of stem cells to the brain. In addition, our data show that topical MSC application proves to be an excellent paradigm to study interactome and reciprocal activation of signaling pathways in transplanted MSC and recipient cerebral cortex in this rat model of TBI.

## Materials and Methods

### Adipose-derived mesenchymal stem cells (MSCs)

Mesenchymal stem cells (MSCs) were derived from the adipose tissue of male transgenic Sprague-Dawley (SD) rats expressing Green Fluorescent Protein (GFP) (SD-Tg(CAG-EGFP)CZ-0040sb) (SLC Inc., Japan). Briefly, the tissue was washed extensively with sterile phosphate buffered saline (PBS) and then the tissue was treated with 0.1% collagenase (type I; Sigma-Aldrich) in PBS for 30 minutes at 37 °C with gentle agitation. After filtration through 100-μm mesh filter to remove debris, the filtrate was washed three times and completely suspended in Dulbecco’s modified Eagle’s medium supplemented with 10% fetal bovine serum, 100 units/ml penicillin, 100 μg/ml streptomycin, and 2 mM L-glutamine. The cultures were maintained in an incubator with a humidified atmosphere of 5% CO_2_.

The cell phenotype of MSCs was tested by flow cytometer with a FACScan argon laser (BD Bioscience, San Jose, CA). MSC suspension was washed and stained with phycoerythrin-conjugated antibodies (red fluorescence) against CD45 and CD90 (Abcam Inc., Cambridge, UK) and CD 29 (Biolegend, San Diego, CA). Isotype-matched negative controls were used to assess background fluorescence.

#### Adipogenic, Chondrogenic and Osteogenic differentiation potential

MSCs were cultured in adipogenic, chondrogenic, and osteogenic differentiation culture media according to the manufacturer’s protocols (Invitrogen, Life TechnologiesTM). The differentiated adipocytes were stained with Oil Red O, chondrocytes with Alcian Blue, and osteocytes with Alizarin Red S stain to identify intracytoplasmic lipid, extracellular glycosaminoglycans, and calcium deposits, respectively.

#### Experimental Traumatic brain injury (TBI) in rats and topical MSC transplantation

All procedures involving animals were conducted in accordance with the guidelines of the Animals (Control of Experiments) Ordinance Chapter 340, Department of Health, Hong Kong, and the study was approved by the Animal Experimentation Ethnics Committee of the Chinese University of Hong Kong. Male, wild-type Sprague Dawley (SD) rats (250–300 gm) were anesthetized with intraperitoneal administration of ketamine (125 mg/kg) and placed on a stereotactic frame. A midline cranial incision was made to expose the skull. On the right side of hemisphere, a circular (5 mm) craniotomy was performed between bregma and lambda and 1 mm lateral to the midline using a dental micro-drill. TBI was induced over the exposed right parietal cortex (n = 20) by impacting a 3-mm diameter tip of an electromagnetic controlled cortical impact (CCI) device at a rate of 4 m/s and 2.5 mm of compression^27^. Then the dura (3 mm × 3 mm) was removed and 1.5 × 106 MSCs were applied onto the surface of the exposed cortex that was 1 mm away from the circumference of the TBI site. A thin layer of fibrin (TisseelR, Baxter, Switzerland) was applied to fix the cells in position. In the TBI control group (N = 20), the dura was removed and no treatment was given except one drop of PBS was added. During the experiment, rectal temperature of the animals was maintained at 37 °C with a warming pad. Ointment to protect vision was applied to their eyes.

### Behavioral Tests

Motor coordination and balance function after TBI were tested on a linearly accelerating Rotor-RodTM at days 0, 3 7, 10. Average time the animals remained on the rotating rod was recorded. The spatial learning and memory were assessed with Morris Water Maze (Nodulus) daily up to day 14. The pool, six feet in diameter, was divided into equal quadrants. The time for the rats spent to identify the platform hidden below the surface of the pool was recorded. The hidden platform was removed at day 14; a memory (probe) test was conducted to assess the ability of rats to find the quadrant where the platform was previously located. At day 4, the gait analysis was performed with CatWalkTM XT (Nodulus) which is a comprehensive system to assess locomotion deficits. Twenty animals per group were used for behavioral assessment. The original sample size calculation was based on pilot Morris water maze studies.

### Preparation of tissue sections; Immunohistochemistry and Immunofluorescence staining

Seven, seven and six animals of both MSC treatment and control groups were sacrificed at days 3, 7, 14 respectively. Brain tissue of all animals was removed, fixed in 10% and was processed for paraffin-embedded sections for histological examinations and immunostaining evaluation. The MSC trafficking was studied with immunohistochemistry using anti-GFP (Abcam). For the determination of *in vivo* differentiation, MSCs from wild-type SD rats were pre-labeled with CM-DIL (Life Technologies) and the co-expression of red fluorescence with FITC conjugated anti-GFAP, anti-Nestin (Santa Cruz) and anti-NeuN (Millipore) was examined using immunofluorescence staining. Apoptosis staining was done according to manufacturer’s instructions (Roche Diagnostics). Immunohistochemistry staining on paraffin sections using anti-GFP, anti-GFAP, anti-Iba1 (Abcam), PCNA (Wako) was performed according to standard procedures. The neuronal death was studied with Cresyl Violet staining. The expression of chemokine receptor, CXCR4 and stromal SDF-1 (Abcam) were studied using immunofluorescent staining on paraffin sections.

### TBI biomarker Tests

For the biomarker analysis, six additional animals were sacrificed at days 1 and 28 from both TBI with MSC treatment and TBI control groups. Frozen cortical tissues were pulverized with mortar and pestle to a fine powder, and then subjected to lysis buffer extraction (1% Triton X-100, 20 mM Tris-HCl (pH 7.4), 5 mM EGTA, 1 mM dithiothreitol, 1x protease and phosphatase inhibitor cocktail) as described previously^28^ Tissue lysates (20 ug protein) were subjected to SDS-PAGE and immunblotting to assess neuronal [αII-spectrin and spectrin breakdown products (SBDPs)] and gliosis (GFAP) and glial cell injury (GFAP and its BDPs)^29^ Blots were probed with primary antibodies [mouse anti-αII-spectrin (Biomol) or rabbit anti-GFAP (Abcam) at 1:2000 dilution in TBST-5% non-fat milk). After TBST washings, blots were incubated with alkaline phosphatase-conjugated secondary antibody (goat anti-rabbit or anti-mouse IgG) (Novagen) for 1–2 hours, and washed blots were then developed with substrate BCIP-NBT reagent (KPL). Carbonic anhydrase–II (CA-II), a housekeeping protein, was also probed as sample loading controls using a rabbit antibody (Abcam). Quantitative evaluation of target protein band intensities was then done via computer-assisted densitometric scanning (Epson XL3500 scanner) and ImageJ software (version 1.6) (NIH) quantification.

### Gene Expression Microarray Labeling and Hybridization

RNA was extracted from the layer of MSCs growing on the surface of TBI brain (N = 3) and normal brain (N = 3) 3 days after topical application. The extracted samples were hybridized with Agilent 4 × 44 K Rat Gene Expression Microarray according to manufacturer protocol.

The data were analyzed with GeneSpring GX 12.5 (Agilent Technologies, Santa Clara, CA) for 75 percentile shift and baseline to median of all samples normalization according to the guided workflow. The gene expression of ‘MSC population on TBI brain’ with fold change ≥2 and p-value ≤ 0.05 were compared with ‘MSC population on normal brain’ using “control MSC pellet” as reference.

### PCR array analysis

84 genes related to neurogenesis were studied using RT2 ProfilerTM PCR Array (Qiagen). At day 3, RNA was extracted from penumbral cortex from TBI injured (N = 4) or uninjured (N = 4) rats, empirically amplified and undergone RT-PCR using commercial available primer-sets according to manufacturer’s protocol.

### Statistical analysis

Numerical data were expressed as mean ± standard deviation. The histological data and RT-PCR were evaluated statistically using IBM SPSS Statistics (Version 22). Differences in the behavioral results and TBI biomarkers were analyzed with the group- based ANOVA and pair-wise Tukey test. Micro-array data were studied using moderate T-test. PCR array results were exported to an Excel file to create a table of CT values which were then uploaded on to the data analysis web portal (http://www.quiagen.com/genelobe). The difference was considered significant if p < 0.05.
